# Chemotherapy and adverse cardiovascular events in colorectal cancer patients undergoing surgical resection

**DOI:** 10.1186/s12957-021-02125-5

**Published:** 2021-01-21

**Authors:** Chieh Yang Koo, Bee-Choo Tai, Dedrick Kok Hong Chan, Li Ling Tan, Ker Kan Tan, Chi-Hang Lee

**Affiliations:** 1 Department of Cardiology, National University Heart Centre Singapore, 1E Kent Ridge Road, NUHS Tower Block Level 9, Singapore, 119228 Singapore; 2 Saw Swee Hock School of Public Health, National University of Singapore, Singapore, Singapore; 3 Division of Colorectal Surgery, University Surgical Cluster, National University Health System Singapore, Singapore, Singapore

**Keywords:** Cardio-toxicity, Cancer survivors, Coronary artery disease, Cardio-oncology, Preventive medicine

## Abstract

**Abstract:**

**Background:**

Colorectal cancer patients undergoing surgical resection are at increased short-term risk of post-operative adverse events. However, specific predictors for long-term major adverse cardiac and cerebrovascular events (MACCE) are unclear. We hypothesised that patients who receive chemotherapy are at higher risk of MACCE than those who did not.

**Methods:**

In this retrospective study, 412 patients who underwent surgical resection for newly diagnosed colorectal cancer from January 2013 to April 2015 were grouped according to chemotherapy status. MACCE was defined as a composite of cardiovascular death, myocardial infarction, stroke, unplanned revascularisation, hospitalisation for heart failure or angina. Predictors of MACCE were identified using competing risks regression, with non-cardiovascular death a competing risk.

**Results:**

There were 200 patients in the chemotherapy group and 212 patients in the non-chemotherapy group. The overall prevalence of prior cardiovascular disease was 20.9%. Over a median follow-up duration of 5.1 years from diagnosis, the incidence of MACCE was 13.3%. Diabetes mellitus and prior cardiovascular disease were associated with an increased risk of MACCE (subdistribution hazard ratio, 2.56; 95% CI, 1.48-4.42) and 2.38 (95% CI, 1.36-4.18) respectively. The chemotherapy group was associated with a lower risk of MACCE (subdistribution hazard ratio, 0.37; 95% CI, 0.19-0.75) compared to the non-chemotherapy group.

**Conclusions:**

Amongst colorectal cancer patients undergoing surgical resection, there was a high incidence of MACCE. Diabetes mellitus and prior cardiovascular disease were associated with an increased risk of MACCE. Chemotherapy was associated with a lower risk of MACCE, but further research is required to clarify this association.

## Introduction

As colorectal cancer survival continues to improve, the burden of cardiovascular disease has similarly increased amongst survivors [1]. Colorectal cancer patients are associated with an increased risk of developing cardiovascular disease and heart failure compared to non-cancer controls [2]. The risk of myocardial infarction and stroke specifically has been reported to be twofold higher than in non-cancer controls [3]. This elevated risk also extends to colorectal cancer patients undergoing surgical resection, where such patients are at increased risk of short-term myocardial infarction and mortality [4, 5].

Colorectal cancer patients are potentially at high cardiovascular risk when undergoing colorectal surgery. This is due to the presence of multiple shared cardiovascular risk factors, chronic inflammation from cancer, and well-established cardio-toxic effects of the chemotherapy regimens comprising of anti-metabolite regimens such as 5-fluorouracil or its pro-drug capecitabine [6]. Although recent guidelines have attempted to address cardiovascular care during cancer treatment, there remains no specific guidance for the patient undergoing cancer surgery [7]. Existing studies examining post-operative outcomes were either not specific to colorectal cancer surgery, or focused mainly on short-term mortality outcomes. These studies also did not account for the potential effect of chemotherapy on outcomes [4, 5].

As such, our objectives were to identify risk factors predictive of major adverse cardiac and cerebrovascular events (MACCE) in colorectal cancer patients undergoing surgical resection. We hypothesised that in colorectal cancer patients undergoing surgical resection, those who received chemotherapy would be associated with an increased risk of MACCE than those who did not receive chemotherapy.

## Methods

This retrospective cohort study was conducted at the National University Hospital Singapore, a tertiary university hospital centre. Consecutive patients who underwent surgery for colorectal cancer from January 2013 to April 2015 were included for analysis. Study participants were observed until the diagnosis of death or until the last day of follow-up on 31 May 2019. This study was approved by the local ethics review board (Domain Specific Review Board-C, National Healthcare Group 2017/00631).

### Data collection

Individual patient-level data was collected via the hospital medical electronic health records system. Demographic characteristics collected included age at time of cancer diagnosis, sex, ethnicity, body mass index and family history of cancer. Cardiovascular risk factors such as smoking status, hypertension, hyperlipidaemia, diabetes mellitus and family history of cardiovascular disease were recorded. Details on relevant medical co-morbidities such as atrial fibrillation, prior cardiovascular disease, peripheral arterial disease, chronic kidney disease and current medications were collected. The Charlson co-morbidity index was calculated for each patient. Cancer characteristics including site, grade, staging, histology and the use of radiotherapy or chemotherapy were collected.

### End points

Major adverse cardiac and cerebrovascular events (MACCE) was defined a priori as a composite of cardiac death, non-fatal myocardial infarction, non-fatal stroke or transient ischaemic attack, unplanned revascularisation, hospitalisation for heart failure and hospitalisation for unstable angina. Other endpoints of interest included new-onset atrial fibrillation and non-cardiac death. All events were defined according to the Standardised Data Collection for Cardiovascular Trials Initiative [8]. Cardiovascular disease was defined as the presence of ischaemic heart disease, stroke or peripheral arterial disease based on electronic records. Clinical event data was collected by a member of the research team blinded to the patient’s cancer and chemotherapy status. These were further adjudicated by a separate member of the team similarly blinded to the patient’s cancer and chemotherapy status.

### Statistical analysis

The patients were divided according to their chemotherapy status. Those who received neoadjuvant or adjuvant chemotherapy were assigned to the chemotherapy group, and those who did not receive chemotherapy at any stage of treatment were assigned to the non-chemotherapy group. Categorical risk factors were summarised using frequencies and percentages, and compared between the two groups via the Fisher’s exact test. Continuous covariates such as age and body mass index were summarised using mean and standard deviation and compared using the Student’s *t* test. Cumulative incidence curves were constructed and compared between the chemotherapy and non-chemotherapy groups using the competing risks method for MACCE [9], accounting for non-cardiovascular death as a competing risk, with adjustment for potential confounders made using the competing risks regression [10]. All analyses were generated using STATA v. 16 (StataCorp LP, College Station, TX, USA), assuming a two-sided test at a 5% level of significance.

## Results

### Baseline characteristics

Between January 2013 and April 2015, data from 412 eligible patients (mean age 64.9 years) were analysed. There were 200 patients who received chemotherapy (chemotherapy group) and 212 patients who did not receive chemotherapy (non-chemotherapy group). The chemotherapy group was significantly younger than the non-chemotherapy group. The baseline demographics and clinical characteristics are shown in Table 1.

**Table 1:** Patient demographics and clinical characteristics

Characteristics	Total (***n*** = 412)	Chemotherapy (***n*** = 200)	No chemotherapy (***n*** = 212)	***P*** value
**Demographics**				
Age in years, mean (SD)	64.9 (11.2)	60.54 (9.7)	69.1 (11.0)	< 0.001
Male sex (*n*, %)	209 (50.7)	103 (51.5)	106 (50.0)	0.761
Ethnicity (*n*, %)				0.747
Chinese	332 (80.6)	157 (78.5)	175 (82.6)	
Malay	59 (14.3)	31 (15.5)	28 (13.2)	
Indian	12 (2.9)	7 (3.5)	5 (2.4)	
Others	9 (2.2)	5 (2.5)	4 (1.9)	
**Anthropometrics, mean (SD)**				
Body mass index-baseline (kg/m^2^)	23.5 (4.4)	23.9 (0.3)	23.2 (0.3)	0.096
**Cardiovascular risk factors (*n*, %)**				
Smoking	46 (11.2)	22 (11.0)	24 (11.3)	0.918
Hypertension	246 (59.7)	102 (51.0)	144 (67.9)	< 0.001
Diabetes mellitus	115 (27.9)	49 (24.5)	66 (31.3)	0.134
HbA1c, %, mean (SD)		6.9 (1.5)	6.8 (1.6)	0.591
Hyperlipidaemia	204 (49.5)	90 (45.0)	114 (53.8)	0.075
Any cardiovascular risk factor (*n*, %)	312 (75.7)	139 (69.5)	173 (81.6)	0.004
No. of cardiovascular risk factor (*n*, %)				0.007
0	100 (24.3)	61 (30.5)	39 (18.4)	
1	8 (1.9)	5 (2.5)	3 (1.4)	
2	35 (8.5)	21 (10.5)	14 (6.6)	
3	223 (54.1)	91 (45.5)	132 (62.3)	
4	46 (11.2)	22 (11.0)	24 (11.3)	
**Family history of cancer (*n*, %)**	68 (16.5)	48 (24.0)	20 (9.4)	< 0.001
**Family history of colon cancer (*n*, %)**	29 (7.0)	16 (8.0)	13 (6.1)	0.459
**Family history of other cancer (*n*, %)**	28 (6.8)	12 (6.0)	16 (7.6)	0.533
**Charlson co-morbidity index (*n*, %)**				< 0.001
≤ 3	80 (19.4)	54 (27.0)	26 (12.3)	
4-6	252 (61.2)	97 (48.5)	155 (73.1)	
≥ 7	80 (19.4)	49 (24.5)	31 (14.6)	
**Concomitant conditions (*n*, %)**				
Prior cardiovascular disease	86 (20.9)	31 (15.5)	55 (25.9)	0.009
Previous myocardial infarction	29 (7.0)	9 (4.5)	20 (9.4)	0.050
Previous percutaneous coronary intervention	25 (6.1)	10 (5.0)	15 (7.8)	0.378
Previous coronary artery bypass grafting	11 (2.7)	5 (2.5)	6 (2.8)	0.835
Stroke/transient ischaemic attack	24 (5.8)	4 (2.0)	20 (9.4)	0.001
Peripheral artery disease	12 (2.9)	3 (1.5)	9 (4.3)	0.098
Atrial fibrillation	18 (4.3)	5 (2.5)	13 (6.1)	0.071
Chronic kidney disease	16 (3.9)	2 (1.0)	14 (6.6)	0.003
**Cancer characteristics**				
**Site (*n*, %)**				0.004
Caecal	20 (4.9)	7 (3.5)	13 (6.1)	
Ascending	41 (10.0)	14 (7.0)	27 (12.7)	
Transverse	49 (11.9)	19 (9.5)	30 (14.2)	
Descending	36 (8.7)	20 (10.0)	16 (7.6)	
Sigmoid	140 (34.0)	67 (33.5)	73 (34.4)	
Rectosigmoid	38 (9.2)	15 (7.5)	23 (10.9)	
Rectum	88 (21.4)	58 (29.0)	30 (14.2)	
**T stage (*n*, %)**				< 0.001
1	38 (9.2)	7 (3.5)	31 (14.6)	
2	41 (10.0)	18 (9.0)	23 (10.9)	
3	221 (53.6)	103 (51.5)	118 (55.7)	
4	112 (27.2)	72 (36.0)	40 (18.9)	
**N stage (*n*, %)**				< 0.001
0	229 (55.6)	58 (29.0)	171 (80.7)	
1	129 (31.3)	96 (48.0)	33 (15.6)	
2	54 (13.1)	46 (23.0)	8 (3.8)	
3				
**M stage (*n*, %)**				< 0.001
0	353 (85.7)	149 (74.5)	204 (96.2)	
1	59 (14.3)	51 (25.5)	8 (3.8)	
**Stage (*n*, %)**				< 0.001
1	64 (15.5)	11 (5.5)	53 (25.0)	
2	155 (37.6)	39 (19.5)	116 (54.7)	
3	134 (32.5)	99 (49.5)	35 (16.5)	
4	59 (14.3)	51 (25.5)	8 (3.8)	
**Histological cell type (*n*, %)**				0.460
Adenocarcinoma	399 (96.8)	195 (97.5)	204 (96.2)	
Others	13 (3.2)	5 (2.5)	8 (3.8)	
**Grade (*n*, %)**				0.038
Low/well differentiated	29 (7.0)	11 (5.5)	18 (8.5)	
Moderate	356 (86.4)	170 (85.0)	186 (87.7)	
Poor/high	27 (6.6)	19 (9.5)	8 (3.8)	
**Margin (*n*, %)**				0.025
Positive	11 (2.7)	9 (4.5)	2 (0.9)	
Negative	401 (97.3)	191 (95.5)	210 (99.1)	
**Radiotherapy (*n*, %)**	55 (13.4)	53 (26.5)	2 (0.9)	< 0.001

*HbA1c* haemogloblin A1c; *M* metastases; *N* nodes; *SD* standard deviation; *T* tumour

In the overall cohort, 65.3% of patients had at least three cardiovascular risk factors. The chemotherapy group had a lower overall prevalence of cardiovascular risk factors, and a lower prevalence of hypertension than the non-chemotherapy group. The prevalence of diabetes in both groups was similar, and the mean HbA1c was 6.94% in the chemotherapy group and 6.80% in the non-chemotherapy group (*p* = 0.59). The chemotherapy group had a lower Charlson comorbidity index score than the non-chemotherapy group. The prevalence of prior cardiovascular disease within the overall cohort was more than 20%, and was significantly lower in the chemotherapy group. Fewer patients in the chemotherapy group had a prior myocardial infarction or stroke, and chronic kidney disease.

### Cancer and clinical details

The oncological details are listed in Table 1. The chemotherapy group had a higher tumour, node, and metastasis stage, and a higher overall tumour-node-metastasis (TNM) stage accordingly. The chemotherapy group had a higher tumour histological grade and positive resection margins. Approximately more than 10% of patients received radiotherapy. Within the chemotherapy group, 77.2% of patients received capecitabine-based regimens whilst the remaining 22.8% received 5-fluorouracil-based regimens.

The medications prescribed at discharge are listed in Table 2. Fewer patients in the chemotherapy group were prescribed with aspirin, angiotensin-converting enzyme inhibitors or angiotensin II receptor blockers. However, there was no significant difference in anti-coagulation, beta-blockers or statins prescribed between the two groups. There was no difference in diabetic medications prescribed between the two groups.

**Table 2:** Discharge medications

Characteristics	Total (***n*** = 412)	Chemotherapy (***n*** = 200)	No chemotherapy (***n*** = 212)	***P*** value
Aspirin (*n*, %)	15 (14.1)	20 (10.0)	38 (17.9)	0.021
Thienopyridines	20 (4.9)	6 (3.0)	14 (6.6)	0.089
Anti-coagulant	9 (2.2)	5 (2.5)	4 (1.9)	0.670
Beta-blocker	101 (24.5)	44 (22.0)	57 (26.9)	0.249
ACE-I/ARB	117 (28.4)	47 (23.5)	70 (33.0)	0.032
Statin	173 (42.0)	75 (37.5)	98 (46.2)	0.073
Metformin	74 (18.0)	33 (16.5)	41 (19.3)	0.453
Sulphonylurea	40 (9.7)	21 (10.5)	19 (9.0)	0.598
DPP-4 inhibitors	9 (2.2)	6 (3.0)	3 (1.4)	0.271
Insulin	15 (3.6)	4 (2.0)	11 (5.2)	0.084

*ACE-I* angiotensin-converting enzyme inhibitors; *ARB* angiotensin II receptor blockers; *DPP-4* dipeptidyl peptidase-4

### Follow-up and clinical outcomes

**Fig. 1:**
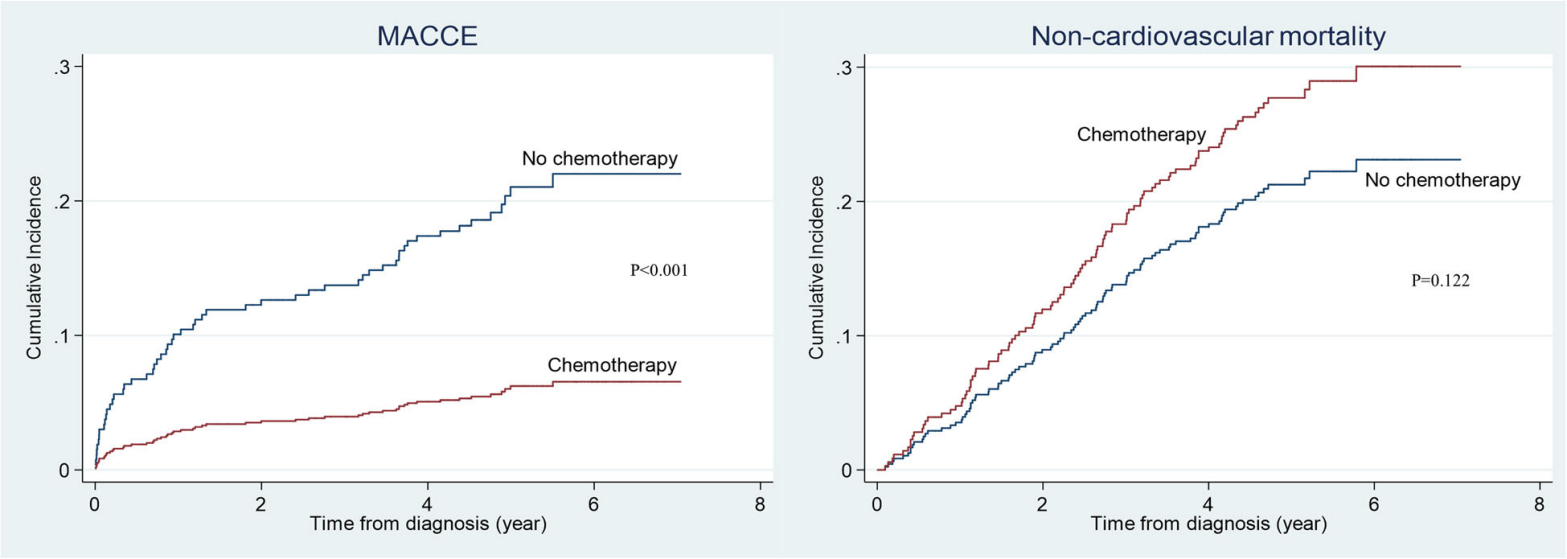
Cumulative incidence of MACCE and non-cardiovascular mortality. Kaplan-Meier curves showing the cumulative incidence of MACCE and non-cardiovascular mortality in the chemotherapy group (red) and the non-chemotherapy group (blue). MACCE, major adverse cardiac and cerebrovascular events

Over a median follow-up of 5.1 years, 55 patients (13.3%) experienced MACCE, including 6 cardiovascular deaths, 29 non-fatal myocardial infarctions, 13 non-fatal strokes, 7 unplanned revascularisations, 13 hospitalisations for heart failure, and 9 hospitalisations for unstable angina (Table 3). The cumulative incidence of MACCE is shown in Fig. 1. The crude cumulative incidence of MACCE was significantly lower in the chemotherapy group than the non-chemotherapy group (3-year estimate, 4.0% versus 13.7%; *p* < 0.001). The crude cumulative incidence for non-cardiovascular death was higher in the chemotherapy group than the non-chemotherapy group, though it did not reach statistical significance (3-year estimate, 12.7% versus 19.5%; *p* = 0.122). The majority of non-cardiovascular deaths were attributed to death from the index cancer. There was no difference between the groups in terms of incident new atrial fibrillation. As part of an exploratory analysis, after excluding all patients with prior cardiovascular disease (*n* = 86), the crude cumulative incidence of MACCE remained lower in the chemotherapy group (3-year estimate 3.0% versus 8.0%; *p* = 0.005).

**Table 3:** MACCE events and other outcomes of interest

Characteristics	Total (***n*** = 412)	Chemotherapy (***n*** = 200)	No chemotherapy (***n*** = 212)
MACCE	**55**	**12**	**43**
Cardiovascular mortality	6	1	5
Non-fatal myocardial infarction	29	6	23
Non-fatal stroke	13	3	10
Unplanned revascularisation	7	0	7
Hospitalisation for heart failure	13	1	12
Hospitalisation for unstable angina	9	2	7
Non-cardiovascular death	102	56	46
New atrial fibrillation	18	5	13

*MACCE* major adverse cardiac and cerebrovascular events

Post-operatively, there was no significant difference in complications including major bleeding, anastomotic leak or re-operation between the two groups. There was also no significant difference in post-operative glucose concentration between the two groups. The mean post-operative glucose concentration was 8.82 ± 2.57 mmol/L in the chemotherapy group and 8.91 ± 2.54 mmol/L in the no chemotherapy group (*p* = 0.755).

Through bivariate analysis as shown in Table 4, age, hypertension, diabetes mellitus, hyperlipidaemia, prior cardiovascular disease, peripheral arterial disease, chronic kidney disease, greater number of cardiovascular risk factors and a higher Charlson co-morbidity index were significantly associated with an increased incidence of MACCE. The use of chemotherapy was strongly associated with a lower incidence of MACCE.

**Table 4:** Bivariate analysis of MACCE

Characteristics	SHR	95% CI	***P*** value
**Demographics**			
Age	1.05	1.02-1.07	< 0.001
Male sex	0.85	0.50-1.45	0.554
Ethnicity			0.084
Chinese	Ref		-
Malay	0.47	0.17-1.31	0.150
Indian	1.82	0.63-5.27	0.271
Others	2.79	0.88-8.82	0.081
**Anthropometrics**			
Body mass index (kg/m^2^)	0.99	0.94-1.04	0.619
**Cardiovascular risk factors**			
Smoking	1.57	0.78-3.15	0.208
Hypertension	2.91	1.50-5.62	0.002
Diabetes mellitus	3.17	1.87-5.37	< 0.001
Insulin dependent diabetes	4.66	2.18-9.94	< 0.001
Hyperlipidaemia	2.90	1.60-5.24	< 0001
Any cardiovascular risk factor	4.30	1.54-12.04	0.005
Number of cardiovascular risk factor			0.024
0	Ref		
1	3.20	0.35-29.07	0.302
2	1.39	0.25-7.60	0.704
3	4.68	1.65-13.25	0.004
4	5.12	1.57-16.63	0.007
**Charlson co-morbidity index**			0.064
≤ 3	Ref		
4-6	3.10	1.11-8.66	0.031
≥ 7	3.69	1.22-11.16	0.021
**Concomitant conditions**			
Prior cardiovascular disease	3.55	2.09-6.03	< 0.001
Peripheral artery disease	3.68	1.57-8.67	0.003
Chronic kidney disease	4.58	2.03-10.34	< 0.001
**Cancer risk factors**			
Site of cancer			0.714
Caecal	Ref		
Ascending	1.02	0.32-3.23	0.967
Transverse	0.41	0.11-1.60	0.200
Descending	0.41	0.09-1.78	0.234
Sigmoid	0.70	0.25-1.96	0.493
Rectosigmoid	0.65	0.18-2.32	0.510
Rectum	0.68	0.23-2.01	0.486
T stage			0.842
1	Ref		-
2	1.07	0.37-3.13	0.900
3	0.77	0.32-1.82	0.550
4	0.87	0.34-2.20	0.765
N stage			0.200
0	Ref		
1	0.62	0.33-1.17	0.137
2	0.56	0.22-1.40	0.213
M stage	0.21	0.05-0.87	0.032
**Stage**			0.143
1	Ref		
2	0.95	0.47-1.92	0.895
3	0.74	0.35-1.59	0.451
4	0.19	0.04-0.85	0.030
**Grade**			0.338
Low/well-differentiated	Ref		-
Moderate	0.80	0.33-1.94	0.623
Poor/high	0.20	0.02-1.69	0.141
**Radiotherapy**	0.62	0.25-1.55	0.307
**Chemotherapy**	0.27	0.14-0.51	< 0.001

*MACCE* major adverse cardiac and cerebrovascular events, *Ref* reference, *SHR* subdistribution hazard ratio

Using a competing risks regression model with non-cardiovascular mortality as the competing risk (Table 5), the association between prior cardiovascular disease and diabetes mellitus with MACCE remained statistically significant even after adjusting for age. The presence of diabetes mellitus was associated with the highest risk of MACCE (subdistribution hazard ratio, 2.56; 95% confidence interval, 1.48-4.42). Chemotherapy was conversely associated with a lower risk of MACCE (subdistribution hazard ratio, 0.37; 95% confidence interval 0.19-0.75).

**Table 5:** Significant predictors of MACCE via competing risks regression model

Parameter	OR	95% CI	***P*** value
Chemotherapy	0.37	0.19-0.75	0.006
Prior cardiovascular disease	2.38	1.36-4.18	0.002
Diabetes mellitus	2.56	1.48-4.42	0.001
Age	1.02	0.997-1.05	0.085

*CI* confidence interval, *MACCE* major adverse cardiac and cerebrovascular events, *OR* odds ratio

## Discussion

This study provides insights into the predictors of MACCE in patients with colorectal cancer undergoing surgical resection. There was a high prevalence of prior cardiovascular disease at 20.9%, and the overall incidence of MACCE over a median follow-up period of 5.1 years was high at 13.3%. Diabetes mellitus and prior cardiovascular disease were associated with an increased risk of MACCE (subdistribution hazard ratio, 2.56; 95% confidence interval, 1.48-4.42 and subdistribution hazard ratio 2.38; 95% confidence interval, 1.36-4.18, respectively). After adjusting for non-cardiovascular death as a competing risk, chemotherapy was associated with a lower risk of MACCE.

Diabetes is an established cardiovascular risk factor and is closely associated with adverse cardiovascular events [11]. Diabetes has also been associated with higher risk of mortality in colorectal cancer patients [12]. However, the association of diabetes with peri-operative cardiac events specific to colon cancer resection is mainly equivocal [13, 14]. Prior studies have shown diabetes was associated with both higher and lower short-term mortality, and also lacked longer-term follow-up and cardiovascular outcomes [5, 15–17]. Our results suggest that diabetes is associated with an increased longer-term risk of MACCE post-surgical resection for colorectal cancer. This is consistent with the recognised impact of diabetes on long-term post-operative cardiovascular outcomes [18].

Unlike the expected association of prior cardiovascular disease with an increased risk of MACCE, our finding of chemotherapy being associated with a lower risk of MACCE is admittedly counterintuitive. This is despite the well-recognised association of 5-fluorouracil and capecitabine with ischaemia and coronary vasospasm [6]. Although this finding could be attributed to unidentified confounders, we attempted to adjust for as many variables as possible including non-cardiac death as a competing risk. Nevertheless, some studies have observed a similar trend, including a recent study showing no increase in cardiovascular-related hospitalisations in metastatic colorectal cancer patients with anti-cancer therapy [2, 19, 20]. However, further prospective studies are required to determine the association of chemotherapy with MACCE in this patient population.

Our study results may have several potential clinical implications. Firstly, the high risk of MACCE in an increasing population of colorectal cancer survivors highlights the need for improved primary and secondary cardiovascular disease prevention in a previously neglected patient population [21]. Despite the higher prevalence of cardiovascular risk factors in patients with cancer, these are often sub-optimally controlled [22]. Healthcare providers tend to offer less counselling on health behaviours to cancer survivors [23]. This is also evident within our study population where the use of anti-platelets and statins were far below the observed prevalence of cardiovascular disease. Secondly, specific guidelines for pre-operative cardiac assessment for cancer surgery may be of benefit. Although guidelines for pre-operative cardiac assessment for non-cardiac surgery exist, cardiac assessment in the cancer patient is more complex due to their increased baseline risk of adverse cardiac events, cardio-toxic effects from cancer therapies, as well as the added complexity of any revascularisation in relation to a potentially time-sensitive curative resection [24–26]. Existing pre-operative cardiac risk assessment scores do not account for these variables and hence are not individualised to the patient with colorectal cancer undergoing surgery [13, 27]. Such patients may benefit from a cardio-oncology consult, which has gained recognition in recent years. Cardio-oncology consultation has been beneficial in optimising cardiac function and cancer therapy outcomes, and through a structured pre-operative cardio-oncological assessment prior to cancer surgery may improve post-operative outcomes [28].

The strength of this study is in our longer-term follow-up compared to other studies mainly limited to short-term post-operative outcomes. Moreover, our outcomes on adverse cardiovascular events beyond mortality alone are increasingly important given the improved survival and the high burden of cardiovascular morbidity and mortality within colorectal cancer survivors. Our study also attempted to examine for the effects of chemotherapy on cardiovascular outcomes post-surgery. There are several limitations of our study. First, although we attempted to identify as many potential confounders as possible, there may be unidentified confounders due to the retrospective design of this study. Data such as electrocardiographic or echocardiographic parameters were not available in all patients, although there was no difference in left ventricular ejection fraction between the two groups when comparing available echocardiographic data. Second, data on the route and schedule of 5-fluorouracil administration was not available, which has been shown to vary in incidence of cardio-toxicity [29]. Third, although we collected information on body mass indices, we did not have data available on visceral adiposity and muscle radio-density which have been shown to be associated with increased risk of MACCE in colorectal cancer patients [30]. Lastly, although data on diabetes including medications and glucose concentrations were available, data on diabetic complications was not available which has been associated with adverse post-operative outcomes in colorectal cancer surgery instead of diabetes itself [31].

## Conclusion

In patients with colorectal cancer undergoing surgical resection, there was a high prevalence of cardiovascular risk factors and prior cardiovascular disease. There was a high incidence of MACCE which extended beyond the immediate post-operative period. Diabetes mellitus and prior cardiovascular disease were independently associated with a higher risk of MACCE. Chemotherapy may be associated with a lower risk of MACCE, although prospective studies are required. Further research is needed to better risk stratify and manage colorectal cancer patients undergoing surgical resection to improve cardiovascular outcomes.

## Data Availability

The datasets used and analysed during the current study are available from the corresponding author on reasonable request.
